# Magnetism in iridate heterostructures leveraged by structural distortions

**DOI:** 10.1038/s41598-019-39422-9

**Published:** 2019-03-12

**Authors:** D. Meyers, Yue Cao, G. Fabbris, Neil J. Robinson, Lin Hao, C. Frederick, N. Traynor, J. Yang, Jiaqi Lin, M. H. Upton, D. Casa, Jong-Woo Kim, T. Gog, E. Karapetrova, Yongseong Choi, D. Haskel, P. J. Ryan, Lukas Horak, X. Liu, Jian Liu, M. P. M. Dean

**Affiliations:** 1 Condensed Matter Physics and Materials Science Department, Brookhaven National Laboratory, Upton, New York, 11973 USA; 2 Advanced Photon Source, Argonne National Laboratory, Argonne, Illinois 60439 USA; 3 Department of Physics and Astronomy, University of Tennessee, Knoxville, Tennessee 37996 USA; 4 Beijing National Laboratory for Condensed Matter Physics and Institute of Physics, Chinese Academy of Sciences, Beijing, 100190 China; 5 School of Physical Sciences, University of Chinese Academy of Sciences, Beijing, 100049 China; 6 School of Physical Sciences, Dublin City University, Dublin 9, Ireland; 7 Department of Condensed Matter Physics, Charles University, Ke Karlovu 3, Prague, 12116 Czech Republic; 8 Collaborative Innovation Center of Quantum Matter, Beijing, China

## Abstract

Fundamental control of magnetic coupling through heterostructure morphology is a prerequisite for rational engineering of magnetic ground states. We report the tuning of magnetic interactions in superlattices composed of single and bilayers of SrIrO_3_ inter-spaced with SrTiO_3_ in analogy to the Ruddlesden-Popper series iridates. Magnetic scattering shows predominately *c*-axis antiferromagnetic orientation of the magnetic moments for the bilayer, as in Sr_3_Ir_2_O_7_. However, the magnetic excitation gap, measured by resonant inelastic x-ray scattering, is quite different between the two structures, evidencing a significant change in the stability of the competing magnetic phases. In contrast, the single layer iridate hosts a more bulk-like gap. We find these changes are driven by bending of the *c*-axis Ir-O-Ir bond, which is much weaker in the single layer, and subsequent local environment changes, evidenced through x-ray diffraction and magnetic excitation modeling. Our findings demonstrate how large changes in the magnetic interactions can be tailored and probed in spin-orbit coupled heterostructures by engineering subtle structural modulations.

## Introduction

Atomic scale layering of disparate materials to form an artificial heterostructure is a promising method for the intelligent design of emergent properties^1–3^. Systems incorporating strong spin-orbit coupling are particularly intriguing as candidates for designer magnetic phases due to the coupling of the magnetic moments to structural distortions. Towards this goal, iridates, compounds composed of active Ir 5*d* orbitals in oxygen octahedra, have emerged as an important new class of correlated material^4–13^. The combination of crystal field interactions and strong spin-orbit coupling, *λ*~0.5 eV, generates narrow bands often leading to Mott insulating antiferromagnetic ground states despite the modest values of the Coulomb repulsion *U*~1 eV^5,14,15^. The resulting ${J}_{{\rm{eff}}}=\frac{1}{2}$ pseudospin states are then intimately coupled with the Ir orbitals and thus are much more sensitive to structural changes than pure spin $S=\frac{1}{2}$ moments. This is exemplified by the different magnetic ground states appearing in iridates composed of similar Ir-O octahedra when they are subtly distorted or interspaced with different atoms. For instance, Sr_2_IrO_4_, hosting isolated IrO_2_ layers, forms an *ab*-plane canted antiferromagnetic state^6,16^, while Sr_3_Ir_2_O_7_, hosting isolated IrO_2_ bi-layers, has robust *c*-axis collinear antiferromagnetic ordering stabilized by a 90 meV magnon gap^17,18^. These competing magnetic ground states establish the iridates as ideal candidates for utilizing controlled structural perturbation to rationally engineer the magnetic behavior.

Towards this goal, layered iridate heterostructures, or superlattices (SL), were recently realized via alternating layers of *n*SrIrO_3_ and SrTiO_3_ (*n*SIO/1STO) opening up routes to tailor magnetic phenomena within artificial Ruddlesden-Popper analogues^14,19^. How magnetic couplings change within such heterostructures as compared to their bulk analogues is, however, unknown, with conflicting reports of *c*-axis versus canted *ab*-plane magnetic ground states for *n* = 2^19–21^. The issue is rooted in the challenge of pinpointing and quantifying the various magnetic interactions in ultrathin heterostructures. In this paper, we directly probe the magnetic behavior of *n*SIO/1STO and extend the sensitivity of Ir *L*_3_ resonant inelastic x-ray scattering (RIXS) to quantify the interactions that stabilize this state. While the bulk-like spin-flop transition is preserved, the relative stability of the magnetic ground state is severely perturbed in the bilayer moving this system towards a quantum phase transition. We find structural leveraging through direct interfacial octahedra manipulation destabilizes the magnetic order via the spin-orbit coupling, revealing a promising new route to engineer the magnetic structure and an experimental scheme to determine the outcome.

## Results

### Crystal and magnetic structure

**Figure 1:**
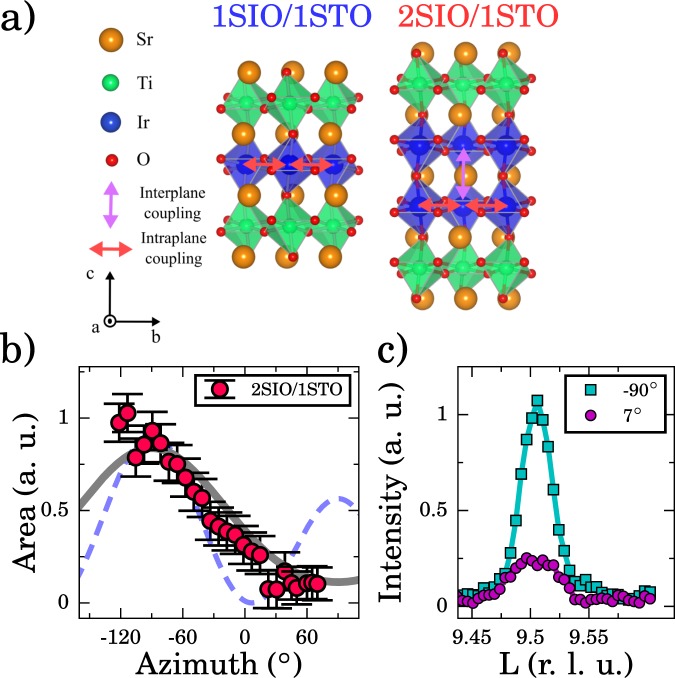
(**a**) Structure of the two SLs. Red arrows show in-plane magnetic exchange pathways and the pink arrow shows the out-of-plane exchange in the bilayer. (**b**) Left panel: Integrated intensity as a function of the azimuthal angle for the (0.5, 0.5, 9.5) magnetic reflection of the 2SIO/1STO sample is shown as red circles, along with the expected response for *c*-axis (solid grey line) and in-plane moment orientations (blue dashed lines). Right panel: Normalized magnetic Bragg peaks at maximum azimuth (90°) and where no intensity is expected for in-plane moments (7°).

Figure 1(a) shows the structure of SLs studied here. SLs of *n*SIO/1STO with *n* = 1, 2, 3 all displayed a net ferromagnetic moment, which was taken as evidence for the stabilization of canted *ab*-plane magnetic moments as in Sr_2_IrO_4_ ^19^. Although there is a strong consensus that this is valid for 1SIO/1STO, the result for 2SIO/1STO is more controversial as it breaks the analogy between *n* = 2 and Sr_3_Ir_2_O_7_, which has c-axis nearly-collinear antiferromagnetism implying only a very small spontaneous net moment^18,19^. The moment seen in 2SIO/1STO was nearly an order of magnitude larger than seen in bulk^22^. This result is quite striking as the magnetic ground state for Sr_3_Ir_2_O_7_ was shown to be very stable for bilayer iridates in general^18,23^. Theory also predicted *c*-axis moments for *n* = 2 and posited that the observed net ferromagnetic moment instead comes from oxygen vacancies^20,24^. Establishing the true magnetic ground state is of high importance towards extracting the magnetic exchange parameters that ultimately dictate the overall magnetic behavior of these heterostructures.

In view of this controversy, we directly measured the spin ordering direction using azimuthal resonant elastic x-ray scattering (REXS) scans, as was done in Sr_3_Ir_2_O_7_ ^23,25^. This dependence is shown for 2SIO/1STO in Fig. 1(b,c). For the magnetic reflection, the SL structure is used for the reciprocal lattice units (r. l. u.), with *a* ≈ *b* ≈ *c*/3. The calculated azimuthal dependence for *c*-axis oriented antiferromagnetic moments, shown as the grey line, matches the data well and establishes predominately *c*-axis moments. Furthermore, the key difference between the two orderings is the 2*π* (*c*-axis oriented) vs. *π* (*ab*-plane canted) angular period of the peaks, with our data clearly only being consistent with the prior. To further emphasize this distinction, we also show the magnetic Bragg peaks where the maximum intensity for both cases is expected (−90°), and also where no intensity for in-plane moments is expected (7°), Fig. 1(b), right panel. Clearly, a magnetic peak persists with integrated intensity that matches that expected for *c*-axis oriented moments (~30%). These results then agree with theoretical predictions, showing the 2SIO/1STO SL maintains the same magnetic ground state as Sr_3_Ir_2_O_7_, strengthening the analogy to Ruddlesden-Popper series iridates^20^. Armed with the correct ground state, the magnetic excitation spectrum can be correctly interpreted, allowing the quantification of the magnetic exchange couplings to unravel the true impact of heterostructuring.

### Quantifying magnetic interactions

**Figure 2:**
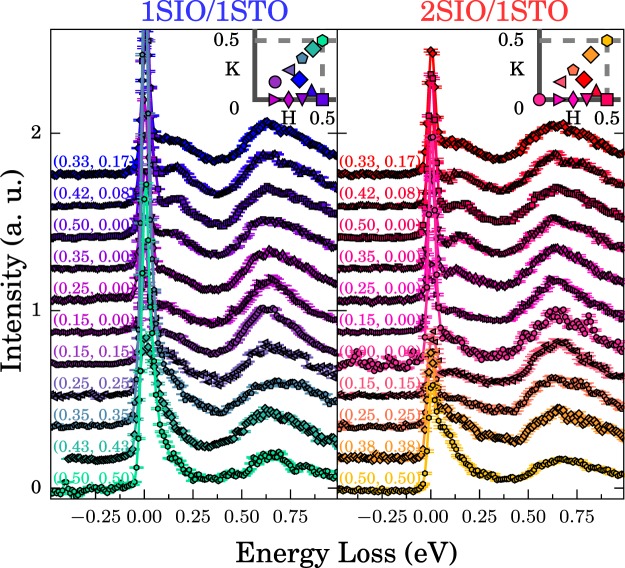
Raw RIXS spectra for 1SIO/1STO (left panel) and 2SIO/1STO (right panel), with measured *Q*-points shown in the inset, where the grey line denotes the Brillouin zone boundary.

**Figure 3:**
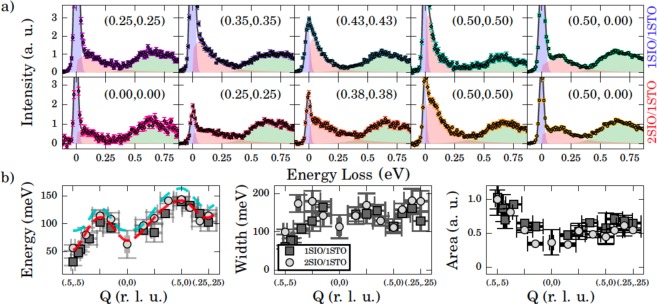
(**a**) RIXS spectra for 1SIO/1STO (top) and 2SIO/1STO (bottom). Spectra colors follow from Fig. 2. The magnon feature is displayed in red, the excitonic features in green, and the elastic line in blue, while the total of these fitting contributions is a dark grey line. (**b**) Extracted energy loss (left), width (middle), and integrated intensity (right) of the magnon feature across reciprocal space. The dashed red (cyan) line is the magnon dispersion fit for 2SIO/1STO (Sr_3_Ir_2_O_7_)^23^. The errors are statistical from the fitting, with the errors carried through the area integration calculation.

To directly probe the magnetic interactions, we utilize RIXS to map the dispersion of magnetic excitations above the established ground state. Here the *J*_eff_ = 1/2 spin-wave spectra, which incorporate the SOC effects, occur below ~0.2 eV followed by features at ~0.6 eV corresponding to *dd*-excitations, as seen previously^13,23,26–33^. Although Ir *L*_3_-edge RIXS has been applied extensively to bulk iridate crystals, a full 2D magnetic dispersion curve has never been characterized on SLs due to the relatively large (5 μm) x-ray penetration depth at the Ir L_3_ edge, with only one report observing a magnetic excitation^32^. This challenge was overcome by growing relatively thick SLs (60 IrO_2_ planes) and working near grazing incidence (1°). Raw RIXS spectra for the SLs are displayed in Fig. 2. Each spectra displays a high energy feature around 0.6 eV energy loss, corresponding to both an intra-*t*_2*g*_ orbital excitation and the *e*−*h* continuum^34^. A sharp peak arises at zero energy due to elastic scattering, along with a very weak phonon feature at around 0.04 eV. The elastic feature is more intense in comparison to single crystal studies due to the large x-ray penetration depth leading to significant diffuse scattering from the STO substrate. Finally, a dispersive feature from 50 to 140 meV is seen in all spectra, and is identified as the magnon excitation, with the higher energy tail including multimagnon excitations^13,23,26,27,31,33^. Only one magnetic excitation was observed for both samples, despite the presence of both optical and acoustic modes for the bilayer. This is due to the intensity dependence of each mode as discussed in the supplemental. The spectra were fit using a combination of peaks in a similar approach to that used previously (see supplemental materials)^23,27,29,34^. Examples of fits along the nodal direction for each sample are displayed in Fig. 3(a). We note that using an alternative magnon line shape returned very similar fitting results. We furthermore measured a second 2SIO/1STO sample which also returned very similar results, see supplemental materials. From these fits one can extract the energy, width, and integrated intensity of the magnetic excitation, Fig. 3(b). The intensity peaks at the magnetic ordering wave vector (0.5, 0.5) and the energy loss is within the bandwidth seen for Sr_2_IrO_4_ and Sr_3_Ir_2_O_7_, corroborating our assignment of the feature as a magnetic excitation^23,26^.

From the extracted magnon dispersion, some important observations are immediately clear: (i) both SLs have nearly identical dispersion around the (0.25, 0.25) and (0.5, 0) points with maxima of ~120 and 150 meV, respectively, (ii) both samples show magnon gaps. The zone boundary dispersion at (0.5, 0) is intriguingly similar for both samples, being much smaller than Sr_2_IrO_4_ and slightly lower than Sr_3_Ir_2_O_7_. This behavior is likely due to the more uniform octahedra present in the heterostructures^4,35,36^. For 1SIO/1STO, a small gap of 31.8 ± 6 meV is found. Spin gaps of a similar size have been discussed for doped/undoped Sr_2_IrO_4_ ^29^. For 2SIO/1STO, a larger gap is defined as 57 ± 5 meV at (0.5, 0.5) where the combination of maximum magnon intensity with only one dominant mode gives a high degree of certainty to the fit, as discussed in the supplemental. The massive difference in the magnon gap of 33 meV at (0.5, 0.5), in comparison to Sr_3_Ir_2_O_7_, evidences a very significant modulation of the magnetic interactions. Confidence intervals up to 99.73% for the (0.5, 0.5) points were obtained for both samples, detailed in the supplemental.

**Figure 4:**
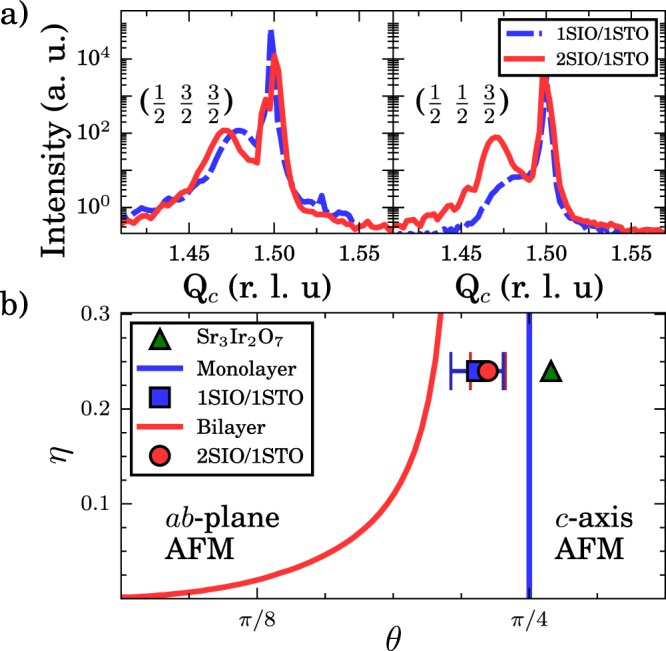
(**a**) Rotation and tilt peaks, left and right panels, for *n* = 1, 2 SLs. Sharp features at Q _*c*_ = 1.5 are substrate reflections, while broad features are SL reflections. (**b**) Classical phase diagram as a function of *η* and *θ*. Phase boundaries between the canted in-plane |*xy*〉 and collinear out-of-plane |*z*〉 orderings are displayed for single layer (blue) and bilayer iridates (red). The position for Sr_3_Ir_2_O_7_ (green triangle) was taken from^23^. The two SLs occupy very similar positions (similar to Sr_2_IrO_4_)^15^, but on different sides of their respective phase transition boundaries, in agreement with the different observed magnetic ground states. Solid lines are the phase boundaries for monolayer (blue) and bilayer (red) iridates. Error bars are propagated from the uncertainty in the magnon energy.

We analyzed the origin of this anomalous behavior using linear spin wave theory (LSWT), which is the leading order term in the expansion of the magnetic excitation spectra where the excitation is taken as a flipped spin distributed throughout the lattice. This was projected to the ${J}_{{\rm{eff}}}=\frac{1}{2}$ states to account for the spin-orbit coupling, as discussed in the supplemental^15,23^. This method was applied to the Hamiltonian described in ref.^23^, with *ab*-plane canted and *c*-axis collinear ground states for the single and bilayer SLs, respectively^23^. Importantly, the canted nature of the moments in 1SIO/1STO gives a dispersion relation that fundamentally differs from the out-of-plane Néel state^23^. As was the case for Sr_3_Ir_2_O_7_, the nine magnetic couplings can be parameterized in terms of: (i) the tetragonal distortion *θ*, defined by $\tan \,2\theta =\frac{2\sqrt{2}\lambda }{\lambda -2{\rm{\Delta }}}$, with spin-orbit coupling *λ* and tetragonal crystal field splitting Δ, (ii) the magnetic bandwidth, $W=\frac{4{t}^{2}}{U}$, (iii) $\eta =\frac{{J}_{H}}{U}$, with Hund’s coupling *J*_*H*_ and Coulomb repulsion *U*, and (iv) the octahedral rotation angle *α*. The form of the exchange couplings in terms of these parameters is described in the supplemental (${J}_{ab}^{^{\prime} }$, ${J}_{ab}^{\text{'}\text{'}}$, and ${J}_{c}^{\text{'}}$ are treated as free fit parameters)^18,23^. Concerning (ii), as shown in Fig. 4(b), the magnetic phase transition is not sensitive to *η* in the relevant regime, nor is the dispersion fit. *η* = 0.24 was established for Sr_3_Ir_2_O_7_ and is unlikely to change significantly, leaving the tetragonal distortion, bandwidth, and rotation angles to explain the observed dispersion and magnon gaps.

Regarding *α*, Sr_2_IrO_4_ and Sr_3_Ir_2_O_7_ both feature large in-plane rotations (*α* = 12° and 11° respectively), but no tilts (Sr_2_IrO_4_) or very small tilts (Sr_3_Ir_2_O_7_ ~1°), with tilts defined as rotations about *a*/*b* axes that bend the *c*-axis bond^4,17,35^. Bulk-like SIO films, on the other hand, show substantial tilts and rotations implying that similar effects may be present in SLs^36^. We consequently tested for the presence of octahedral tilts and rotations by scanning the half order Bragg peak’s locations. While an exact structural solution of the SLs is unfeasible due to the complex orthorhombic structure of SIO^36^, published methods allow us to associate different half order reflections with different antiphase distortions^37–39^. We measured several reflections for the *n* = 1 and 2 samples and illustrate the important behavior in Fig. 4(a). The $(\frac{1}{2},\,\frac{3}{2},\,\frac{3}{2})$ reflection (left panel) arises from a combination of rotations and tilts; whereas the $(\frac{1}{2},\,\frac{1}{2},\,\frac{3}{2})$ reflection (right panel) comes from *only* tilts. Both peaks are of similar magnitude for *n* = 2, but the tilt-peak is suppressed by an order of magnitude in *n* = 1. This data suggests that both SLs have similar rotations of ~8° as found by Matsuno *et al*.^19^. Interestingly, theory results predicted larger rotations of ~14°, but the different values return very similar results in the LSWT analysis and we thus chose to use the experimental values^20^. In contrast with the nearly straight (~1°) *c*-axis bonds seen in Sr_3_Ir_2_O_7_ ^4,17^, *n* = 2 likely hosts tilting of a similar size (of order 8°), while *n* = 1 has small, but finite tilts. Most importantly, the presence of tilting generates the *ab*-plane ferromagnetic moment in 2SIO/1STO, observed experimentally^19,40^, through canting the *c*-axis antiferromagnetic moments, resolving the conflict between previous experimental interpretations and theory for 2SIO/1STO^20,21^. In-plane moments can also be theoretically argued to originate from oxygen vacancies, but such random tilting would give a smaller total moment and a change in Ir valence, not observed here^21^.

### Tuning magnetic ground states

Having established the approximate rotation angles of both samples as *α* = 8°, we can fit the dispersion of the SLs, as discussed above, displayed for 2SIO/1STO in Fig. 3(b) left panel. This allows extraction of the parameterized tetragonal distortion, *θ*, which controls the magnetic phase stability^15,18,23^. In the case of 1SIO/1STO, we find *θ* = (0.226 ± 0.012)*π*^29^. For 2SIO/1STO, we find *θ* = (0.231 ± 0.008)*π*. For bilayer iridates, optical and acoustic modes are present, but only one mode is observed due to the *Q*-dependence of their intensities, discussed in the supplemental. Both of these *θ* values are near to that observed for Sr_2_IrO_4_ (~0.235 *π*), but well below that found for Sr_3_Ir_2_O_7_ (*θ* = (0.26 ± 0.01)*π*), Fig. 4(b) ^17,23,35,36,41^. These results point to the substrate imposed epitaxial structure as the dominant determinant of *θ*, likely due to the identical compressive strain applied to the SIO layers leading to similar local tetragonal distortions, despite the different layering schemes.

Extracted values of the exchange couplings for each of the SLs are shown in Table 1, these are obtained from equations depending only on *θ*, bandwidth *W*, *η*, and *α*. For 1SIO/1STO, the values are similar to those found in both doped and undoped Sr_2_IrO_4_, owing to the relatively small change in *θ* between the 1SIO/1STO and Sr_2_IrO_4_ (~0.01*π*)^29^. This indicates the 1SIO/1STO provides another magnetic analogue to cuprates, similar to that found in Sr_2_IrO_4_, but with the higher tunability afforded by heterostructuring^7,12,42,43^. Comparing 2SIO/1STO with Sr_3_Ir_2_O_7_, on the other hand, the changes are quite substantial, owing to the significant shift of *θ* (0.029*π*) between these two materials^23^. Importantly, a 34% decrease in the pseudodipolar anisotropic coupling Γ_*c*_ is observed, which is chiefly responsible for stabilizing the *c*-axis magnetic ground state. This decrease derives almost exclusively from the smaller magnon gap at (0.5, 0.5), indicative of the destabilization of this *c*-axis anisotropy and consistent with the addition of octahedral tilting in the artificial heterostructure. This is also consistent with the observation of more bulk-like behavior for 1SIO/1STO, where the octahedral tilting is much weaker.

**Table 1:** Exchange parameters (in meV) for *n*SIO/1STO from the best fit of tetragonal distortion *θ*. *J*, Γ and *D* are the Heisenberg, anisotropic and Dzyaloshinskii-Moriya interactions respectively.

*n*	*J*_*ab*_	*J*_*c*_	Γ_*ab*_	Γ_*c*_	*D*_*ab*_	*D*_*c*_	${J}_{ab}^{\text{'}}$	${J}_{ab}^{\text{'}\text{'}}$	${J}_{c}^{\text{'}}$
1	53	—	−0.3	—	11.2	—	−14.2	9.0	—
2	67.0	34.8	−0.6	22.6	14.4	21.7	3.2	9.0	3.1

Subscripts denote coupling directions and ' indicates next neighbor coupling.

To investigate the stability of the observed magnetic phases, we map the SLs on the classical (*θ*, *η*) magnetic phase diagram in Fig. 4(b) alongside Sr_2_IrO_4_ and Sr_3_Ir_2_O_7_ ^18^. Intriguingly, both SLs lie close to their respective phase transitions. This is especially significant for 2SIO/1STO which lies much closer to the phase transition than Sr_3_Ir_2_O_7_, within 0.02*π*, corresponding to a ~10 meV tetragonal splitting change^15^. The fact that such a shift happens despite the similar structures of 2SIO/1STO and Sr_3_Ir_2_O_7_ demonstrates how relatively small distortions can strongly modify the magnetic interactions. Further bending of the *c*-axis bond can thus be expected to drive the system closer to, and eventually through, a quantum critical point between the *ab*-plane canted and *c*-axis antiferromagnetic states. This could be accomplished through applying epitaxial tensile strain by changing the substrate or by substituting Sr with Ca, with the outcome now being observable with RIXS as we demonstrate here. Based on the calculated change in the crystal field of strained films of Sr_2_IrO_4_, applied strain of only a few percent could be enough to drive 2SIO/1SIO to the quantum critical point^20,30^. Importantly, the distortions observed here are ubiquitous for epitaxially strained SLs, which naturally accommodate the imposed lattice parameters through these means. Thus this methodology should be applicable in a wide range of systems. In this way, heterostructuring of systems hosting strong spin-orbit coupling provides a means to exploit small structural distortions to stabilize large changes in the magnetic ground state.

To summarize, utilizing REXS and RIXS we investigated the magnetic structure and interactions in iridate heterostructures and pinpoint their position on the magnetic phase diagram, pushing RIXS to include thin film iridate SLs. We find that the magnetic structures replicate the expected spin-flop transition, but the relative stability of the magnetic phase is strongly perturbed by tilting of the octahedra as evidenced by the reduced magnon gap observed for 2SIO/1STO but not for 1SIO/1STO, which hosts much weaker tilting. Heterostructuring iridates and probing their magnetic interactions with RIXS thus reveals that spin orbit coupling can leverage small structural distortions to potentially dictate the magnetic structure with the additional possibility to engineer quantum critical points.

## Methods

### Sample preparation

SLs of form [*n*SIO/1STO]×*m* with *n* = 1, 2 and *m* = 60, 30, respectively, were grown with pulsed laser deposition using methods described in ref.^40^, as depicted in Fig. 1(a). High sample quality was verified by x-ray diffraction, x-ray magnetic circular dichroism, transport and magnetometry measurements (Figs S1–S3 of the supplemental material), consistent with previous studies^19,40^. Reflectively measurements and SL peaks confirm the *B*-site ordering and lack of stacking faults. Previous measurements of the heterostructures studied here have shown no appreciable deviation of the Ti valance from its 4 + state in bulk STO^40,44,45^. The STO layers in *n*SIO/1STO are consequently expected to have a negligible contribution to the magnetism studied here, although such effects have been discussed in other heterostructures^46–48^.

### X-ray measurements

REXS, RIXS, and non-resonant diffraction data were taken at the 6-ID-B, 27-ID-B, and 33-BM-C beamlines of the Advanced Photon Source at Argonne National Laboratory at base temperature (~14 K). The RIXS energy resolution was 35 meV, full width half maximum. All *Q*-points were taken in grazing incidence with nearly 90° scattering to suppress non spin-flip processes. The resolution in reciprocal space was ± 0.072 r.l.u. A mask was used to improve the resolution to 0.12 Å^−1^ (0.018 r.l.u.) for the (0.5, 0.5) and (0, 0) Q-points for both samples. All REXS data were taken with a graphite polarization analyzer to minimize non-magnetic scattering. Furthermore, both on and off-resonance scans were taken for every azimuth to remove any charge signal leakage and normalize out any extrinsic effects. Further details including analysis and fitting procedures are available in the Supplemental Material.

### Linear spin-wave theory

Linear spin-wave theory was applied to the anisotropic Hamiltonian for the IrO_2_ single and bi-layers as done in previous works^18,23^.

## Supplementary information

Supplemental Materials

## Data Availability

The data that support the findings of this study are available from the corresponding authors upon reasonable request.
