# Supplementary material for: Magnetism in iridate heterostructures leveraged by structural distortions
Source: Sci Rep. 2019 Mar 12;9:4263. doi: 10.1038/s41598-019-39422-9 (PMC6414659; doi:10.1038/s41598-019-39422-9)
Supplement: Supplementary file 1 — Supplemental Materials [file 41598_2019_39422_MOESM1_ESM.pdf]

Supplemental Material: Magnetism in iridate heterostructures leveraged by structural distortions

D. Meyers,[1,*] Yue Cao,[1] G. Fabbris,[1] Neil J. Robinson,[1] Lin Hao,[2] C. Frederick,[2] N. Traynor,[2] J. Yang,[2] J. Q. Lin,[3,4] M. H. Upton,[5] D. Casa,[5] Jong-Woo Kim,[5] T. Gog,[5] E. Karapetrova,[5] Yongseong Choi,[5] D. Haskel,[5] P. J. Ryan,[5,6] Lukas Horak,[7] Xuerong Liu,[3,8] Jian Liu,[2,†] and M. P. M. Dean[1,‡]

[1] *Condensed Matter Physics and Materials Science Department, Brookhaven National Laboratory, Upton, New York 11973, USA*

[2] *Department of Physics and Astronomy, University of Tennessee, Knoxville, Tennessee 37996, USA*

[3] *Beijing National Laboratory for Condensed Matter Physics and Institute of Physics, Chinese Academy of Sciences, Beijing 100190, China*

[4] *School of Physical Sciences, University of Chinese Academy of Sciences, Beijing 100049, China*

[5] *Advanced Photon Source, Argonne National Laboratory, Argonne, Illinois 60439, USA*

[6] *School of Physical Sciences, Dublin City University, Dublin 9, Ireland*

[7] *Department of Condensed Matter Physics, Charles University, Ke Karlovu 3, Prague 12116, Czech Republic*

[8] *Collaborative Innovation Center of Quantum Matter, Beijing, China*

This document provides further details of our sample characterization, experimental setup, data fitting, spin-wave-theory based analysis, and additional measurements of the magnetic interactions.

CONTENTS

I. Sample Characterization 1
  A. Transport and magnetometry 2
  B. XAS and XMCD 2
  C. Reflectivity and Rocking curves 2

II. Further details of experimental methods 3
  A. Resonant elastic x-ray scattering 3
  B. Resonant inelastic x-ray scattering 4
  C. Rotation peak measurements 5

III. Spectra fitting routine 5
  A. Fitting routine 5
  B. Alternative fitting routine 6

IV. Linear spin wave theory analysis 8
  A. Modeling 8
  B. Dispersion Fitting 10
  C. Dimer approach 11

V. Additional data 11
  A. 1SIO/1STO 11
  B. 2SIO/1STO 11

References 13

I. SAMPLE CHARACTERIZATION

In this section we outline our sample characterization which demonstrates the high structural fidelity of the samples and that they show the expected electronic properties consistent with published results [1, 2].

* dmeyers@bnl.gov
† jianliu@utk.edu
‡ mdean@bnl.gov

A. Transport and magnetometry

Electronic transport and SQUID magnetometry measurements are shown in Fig. S1. As can be seen both samples are insulating with weak ferromagnetic signals below their respective T_{Neel}.

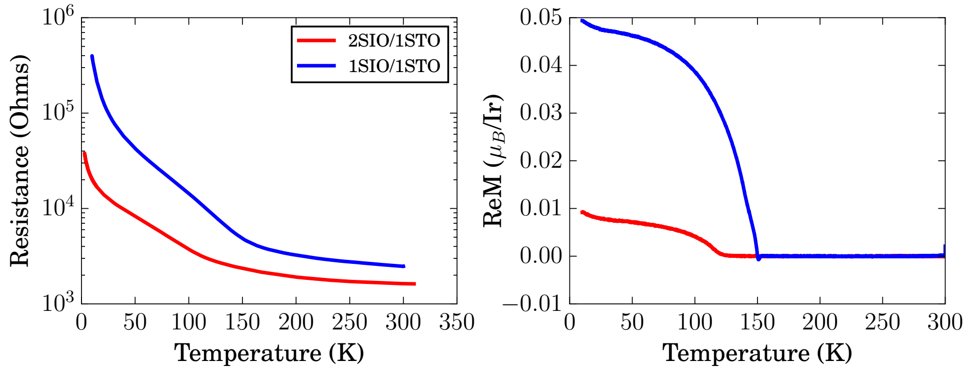

FIG. S1. *ab*-plane electrical transport (left panel) and magnetometry (right panel) measurements on the 1SIO/1STO and 2SIO/1STO SLs [2]. The magnetization measurements show the net *ab*-plane moment measured after cooling with 0.5T applied field.

B. XAS and XMCD

In order to confirm the proper valence and magnetic moments, X-ray absorbtion spectroscopy (XAS) and x-ray magnetic circular dichroism (XMCD) at the Ir $L_{3,2}$-edges were measured at sector 4-ID-D of the Advanced Photon Source at Argonne National Laboratory. The results are displayed in Fig. S2. The samples were cooled to 15K with a basal-plane applied magnetic field of ± 1 T parallel to the x-ray beam. Both samples show lineshapes consistent with the typical Ir^{4+} valence with branching ratios around 6, similar to Sr_2IrO_4 [3]. Utilizing XMCD sum rules, total ordered moments of 0.088 and 0.074 μ_B for $n = 1$ and 2 (respectively) are found, similar to the value found for Sr_2IrO_4 (0.06 μ_B) [4]. These large moments conflict with a dimer-based picture for the magnetic ground state, as a true dimer would have a negligible magnetic moment [5]. Both samples show orbital moments that are much larger then the spin moment, $\langle L_z \rangle = 0.070$ (0.053) and $\langle S_z \rangle = 0.009$ (0.011) for $n = 1$ (2). This behavior, evidenced by the extremely weak L_2 XMCD signal, is typical of the iridates owing to the $J_{\text{eff}} = \frac{1}{2}$ state and leads to the extracted moments being dependent almost entirely on the L_3 XMCD. This also allows us to set an upper limit on the tetragonal splitting, as large splitting will cause a loss of the $J_{\text{eff}} = \frac{1}{2}$ character and appreciable L_2 XMCD [4]. We estimate this upper bound to be approximately 300 meV, giving a maximum θ of 0.25π, well above our fitted values of θ. Angle resolved photo-emission spectroscopy measurements find a similar tetragonal splitting for $Sr_3Ir_2O_7$ [6].

C. Reflectivity and Rocking curves

In order to quantify the interface roughness and confirm the proper sample thickness, reflectivity data for both SLs were fit utilizing GenX [7], displayed in Fig. S3, top panel. For the $n = 1$ sample, the individual layer roughness was found to be 1.25 ± 0.90 Å, with a STO (SIO) c-axis lattice constant of 3.913 ± 0.010 Å (3.960 ± 0.024 Å). For the $n = 2$ sample, the individual layer roughness was found to be 1.96 ± 0.38 Å, with STO (2SIO) c-axis of 3.906 ± 0.010 Å (7.949 ± 0.036 Å). This low interlayer roughness is consistent with high quality SL growth with negligible B-site disorder. Both $n = 1$, 2 samples were found to have the SL repeats corresponding to the RHEED oscillations observed during growth (60, 30 respectively) [2]. Rocking curves are shown in Fig. S3, bottom panel. Films have resolution-limited FWHMs below 0.05°. Further reflection high energy electron diffraction, surface x-ray diffraction data are available in Ref. [2].

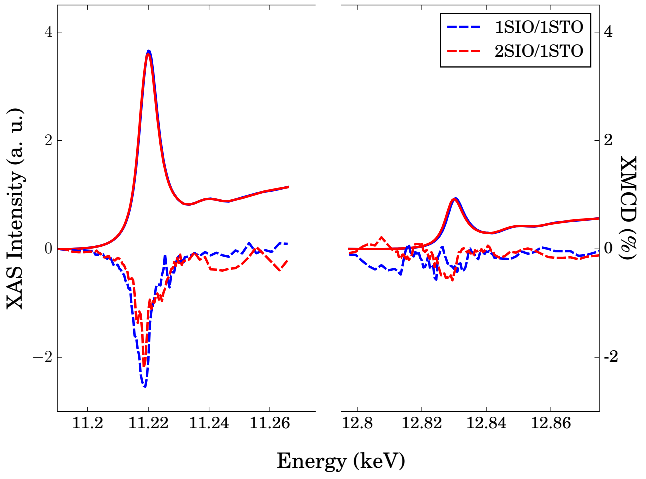

FIG. S2. XAS (left axis) and XMCD (right axis) for Ir $L_{3,2}$ edges (left and right panels respectively).

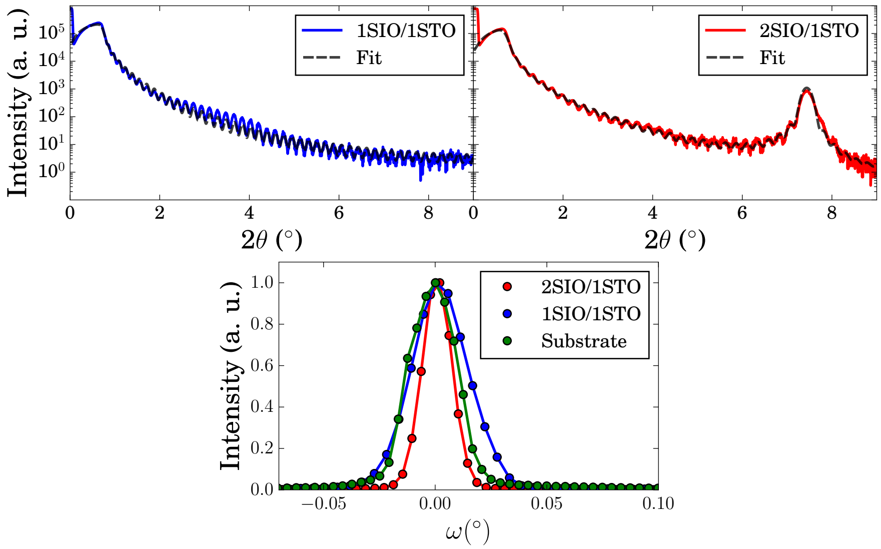

FIG. S3. Top panel: Reflectivity curves with fits for both SLs [2]. Bottom panel: Rocking curves for both SLs along with substrate.

II. FURTHER DETAILS OF EXPERIMENTAL METHODS

A. Resonant elastic x-ray scattering

For the REXS data, azimuthal scans were measured both on (11.215 keV) and off (11.205 keV) resonance. The $[-1\ 1\ 0]$ direction for $SrTiO_3$, in a unit cell with [0 0 1] defined as the sample normal, was taken as the azimuthal reference vector. We used a pesudo tetragonal unit cell with $a = b = 3.905$ Å and $c = 11.91$ Å. The data from 45° to 70° was taken after repositioning the sample to gain access to a larger portion of reciprocal space, due to the limited range of the displex cryostat. Modeling for the different possible ab-plane and c-axis ordering was done using methods described in Refs. [8, 9]. Predominantly ab-plane ordering, shown in Fig. 1(b) of the main text, is the in-plane ordering direction which most closely matches the observed data, while still clearly not showing the proper dependence. For each azimuth, resonant and non-resonant L-scans were taken. The magnetic peak area was taken as the resonant scan area, minus the non-resonant area, divided by the non-resonant scan area to account for any alignment or footprint differences. Extrinsic effects such as footprint and dynamical scattering were ruled out by measuring both on and off resonance. The displayed is taken as the area of the resonance subtracted by the area of the off resonance peak,

divided by the off-resonance signal.

To confirm the magnetic origin of the REXS signal, energy dependence of the (.5 .5 9.5) magnetic reflection were measured, Fig. S4. This distinct resonance profile is well known for the Ir4+ $5d^5$ iridates [10].

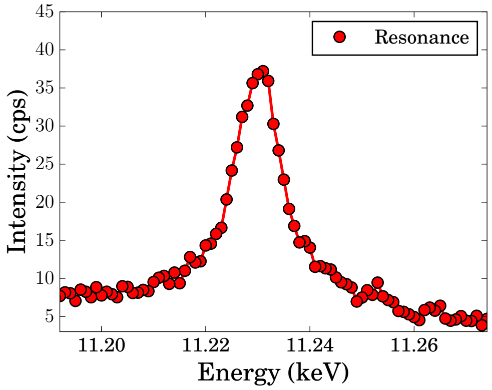

FIG. S4. Resonance of the (0.5 0.5 5.5) magnetic Bragg peak for $n = 2$ SL.

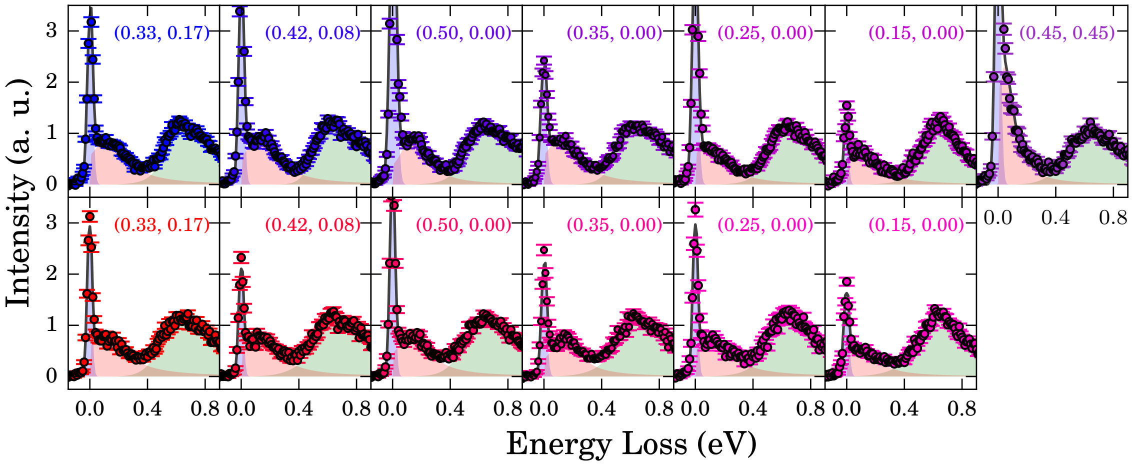

FIG. S5. RIXS Q-points measured for 1SIO/1STO (2SIO/1STO) SL from $(0.25, 0.25)$ to $(0.5, 0)$ and along the zone boundary, top panel (bottom panel).

B. Resonant inelastic x-ray scattering

For RIXS measurements, a diced-Si (8 8 4) analyzer was used at a distance of 2m from the sample. Measurements were undertaken around the (3.5 1.5 10.5) (or the $(0.5, 0.5)$ $Q_{x,y}$-point) magnetic Bragg peak at grazing incidence with nearly 90° scattering when possible to minimize non spin-flip processes. The resolution in reciprocal space was ± 0.072 r.l.u. and ± 0.018 r.l.u. when a $2''$ analyzer mask was used for the samples around the $(0.5, 0.5)$ and (0, 0) points. This was done to better resolve the magnon gap, but the added acquisition time was prohibitive and unnecessary for the majority of Q-space. Q_c was varied to maintain scattering geometry.

The remaining Q-space RIXS data with fits, not shown in Fig. 2 of manuscript, for both samples is displayed in Fig. S5.

C. Rotation peak measurements

Octahedral rotations were measured at 33-BM-C using the Psi-diffractometer. All measurements were done at a base temperature of 10 K using a displex cryostat. For the purposes of comparison a psuedo-cubic unit cell (a×a×a) with just one full O octahedra was used for these measurements and display [11]. In order to rule out any interference effect causing the much smaller intensity for the 1SIO/1STO SL tilt peak we also show the ($\frac{1}{2}$ $\frac{1}{2}$ $\frac{7}{2}$) tilt peak in Fig. S6, where the film-substrate separation is much larger in Q. The rotation peak intensities for both samples were similar and thus we estimated both host approximately 8° rotations as seen in 1SIO/1STO, with a range of $5-15°$ not giving significant changes to the observed θ fitting behavior [1].

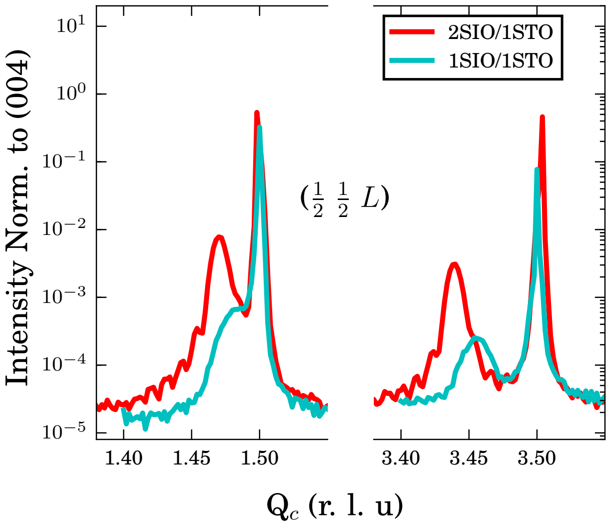

FIG. S6. Two tilt peaks both showing the order of magnitude intensity difference between 1SIO/1STO and 2SIO/1STO.

III. SPECTRA FITTING ROUTINE

A. Fitting routine

Each spectrum was fit using similar methods to those applied to bulk Sr_2IrO_4 and $Sr_3Ir_2O_7$ crystals [12–15]. Four features were used: an elastic Gaussian peak at 0 energy loss, two Gaussians for the orbital excitations around 500 and 700 meV, and a Lorentzian with a Bose temperature factor for the dispersive magnon feature. All of the parameters were allowed to float except the Bose temperature factor, which was set by the experimental measurement temperature (14K). Finally, the width of the higher energy excitonic features were constrained to avoid unphysical broadening (FWHM > 800 meV) .

In Fig. S7, we plot the three Q-points showing the gaps in 2SIO/1STO and 1SIO/1STO. The 2/1 data at $(0.5, 0.5)$ is repeated with a fit where the magnon was forced to be centered at 90 meV, as seen in $Sr_3Ir_2O_7$ [5, 13]. As can be clearly seen, letting all other parameters float fails to obtain a good fit to the data, showing the observed change in magnon gap is independent of fitting scheme. The fitting gave the following values and statistical errors for the magnon position (left to right, excluding the forced fit): 57.0 ± 5.2 meV, 65.9 ± 24.4 meV, and 31.8 ± 6.6 meV. The larger error for the (0, 0) Q-point is due to the expected significant reduction in the magnon intensity that strongly reduces the signal-noise ratio.

For more thorough statistical analysis, confidence intervals were computed for $Q = (0.5,\ 0.5)$ for both samples. For the 2SIO/1STO sample, the (0.5, 0.5) spectra has the following intervals: $52.4-60.6$ meV for 67.4% confidence, $47.0-65.3$ meV for 95.0% confidence, and $41.2-69.5$ meV for 99.7%. Thus, the full 3σ range of values lies at least 20 meV below the bulk $Sr_3Ir_2O_7$ gap. For the 1SIO/1STO sample the following intervals are found : $26.5-36.6$ meV for 67.4% confidence, $20.5-41.1$ meV for 95.0% confidence, and $13.1-45.8$ meV for 99.7%.

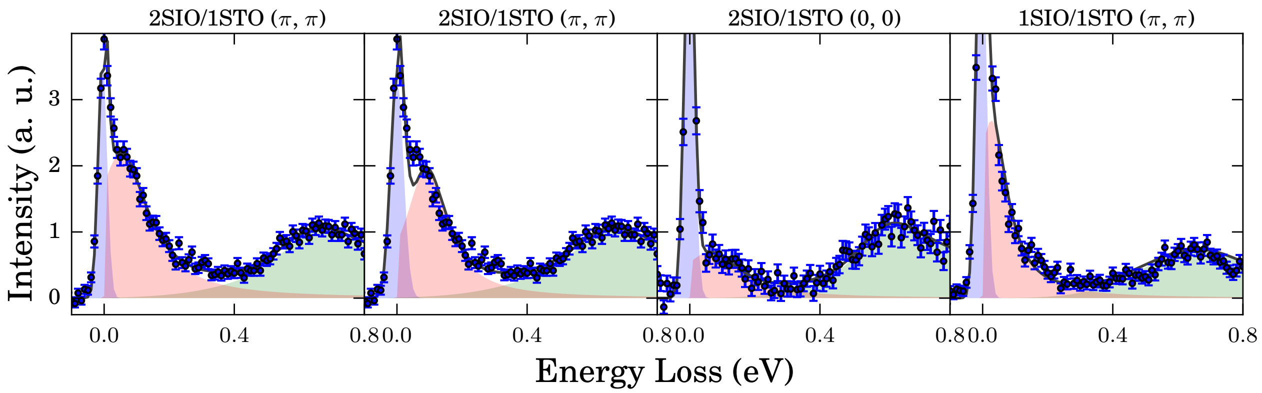

FIG. S7. Fitting curves for the (0.5, 0.5) (2SIO/1STO; left two panels, 1SIO/1STO; far-right panel) and the (0, 0) (2SIO/1STO; middle-right panel) Q-points.

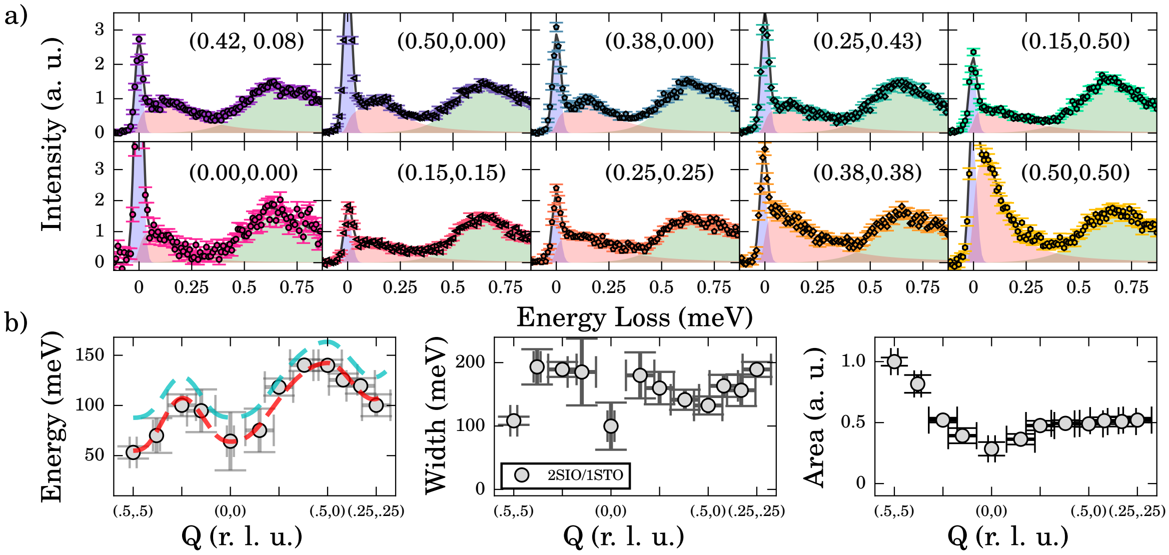

FIG. S8. a) RIXS spectra of 2SIO/1STO with an alternative lineshape fitting. The magnon feature is displayed in red, the excitonic features in green, and the elastic line in blue, while the total of these fitting contributions is a dark grey line. b) Extracted energy loss (left), width (middle), and integrated intensity (right) of the magnon feature across reciprocal space. The dashed red (cyan) line is the magnon dispersion fit ($Sr_3Ir_2O_7$ [13]). The error bars are propagated from the uncertainty in the least-squares fitting routines.

B. Alternative fitting routine

There are several effective methods for fitting the magnetic excitations in RIXS. We present here results for an alternative lineshape for the two samples in the manuscript, along with a second 2SIO/1STO sample, which shows qualitatively very similar results as was found with the above detailed fitting. For this routine a Bose-corrected Lorentzian, as used above, was convoluted with the resolution function and then used to fit the data. Where necessary the elastic line width was frozen to the observed resolution.

In Fig. S8 we show the results of this alternative fitting routine for the 2SIO/1STO sample, as can be seen the dispersion is still clearly visible and very similar to the results in the manuscript. The magnon gap is found to be 53.2±5.0 meV with a 99.7% confidence interval of $37.2 - 65.7$ meV , similar to the 57±5.0 meV found with the other lineshape. Furthermore, extracting the values for θ and W gives $\theta = 0.225 \pm 0.004\pi$ and $W = 13.89 \pm 0.49$ respectively, which is quite similar to that found with the other fitting routine for θ, Fig. S9. Thus the central argument of the manuscript, i.e. the magnon gap is reduced relative to the bulk analogue ($Sr_3Ir_2O_7$) leading to a lowering of θ and thus a reduction in the magnetic phase stability, is supported by this alternative fit routine.

We also applied this alterative fitting routine to the 1SIO/1STO sample, Fig. S10. The results are again quite

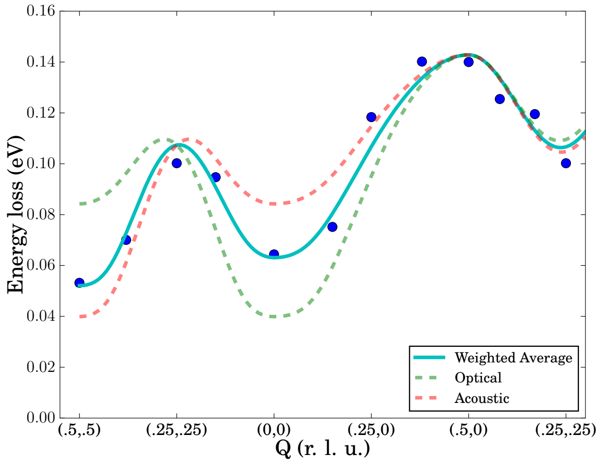

FIG. S9. Fit of the averaged magnon dispersion (cyan line) to that extracted from the RIXS intensity (solid blue points) for the 2SIO/1STO sample with the alternative fitting routine. The optical and acoustic magnon dispersions are calculated within spin wave theory, Eq. (S10), and are averaged to account for their momentum-dependent scattering intensities. Averaging is performed to take into account, to leading order, the energy resolution (35 meV) and weak signal of the experiment.

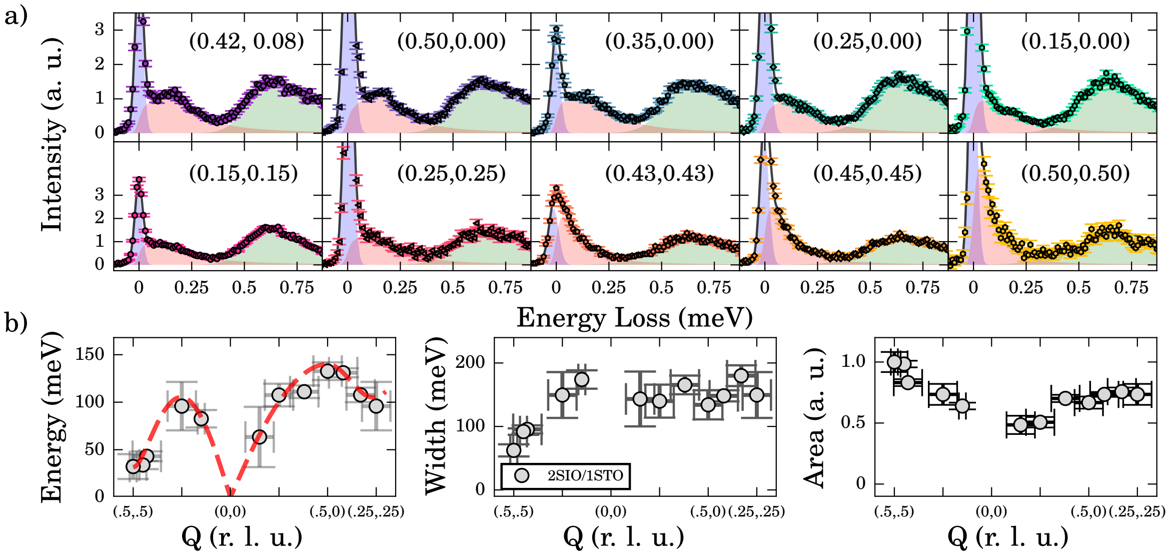

FIG. S10. a) RIXS spectra of 1SIO/1STO with an alternative lineshape fitting. The magnon feature is displayed in red, the excitonic features in green, and the elastic line in blue, while the total of these fitting contributions is a dark grey line. b) Extracted energy loss (left), width (middle), and integrated intensity (right) of the magnon feature across reciprocal space. The dashed red line is the magnon dispersion fit. The error bars are propagated from the uncertainty in the least-squares fitting routines.

similar to what is found with the original fitting routine with a magnon gap of 34±19 meV. The larger error bar here is due to similar energy of the feature to the observed resolution which, when combined with the resolution function convolution and Bose factor, allows the width and height to create similar lineshapes at different positions. However, the spectra at (0.45, 0.45) and (0.43, 0.43) are not as effected and show the obtained gap is reasonable. Extracting the values for θ and W gives $\theta = 0.231 \pm 0.007\pi$ and $W = 22.42 \pm 0.70$ respectively, which is quite similar to that found with the original fitting routine for θ, Fig. S11.

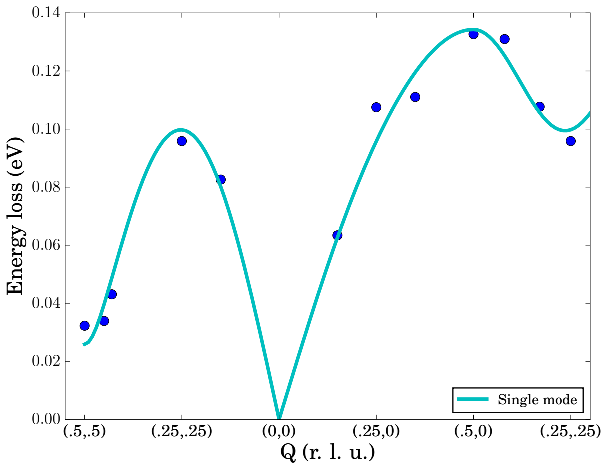

FIG. S11. Fit of the averaged magnon dispersion (cyan line) to that extracted from the RIXS intensity (solid blue points) for 1SIO/1STO with the alternative lineshape.

IV. LINEAR SPIN WAVE THEORY ANALYSIS

A. Modeling

We considered a simple low-energy effective model for these superlattices. The IrO_2 layers lie in the ab-plane, and are vertically stacked in the c-axis. The Hamiltonian is

$$H = H_{ab}^{(1)} + H_{ab}^{(2)} + H_c, \tag{S1}$$

where $H_{ab}^{(l)}$ is the Hamiltonian for each of the IrO_2 planes (labelled by $l = 1, 2$)

$$\begin{aligned} H_{ab}^{(l)} = & \sum_{\langle \vec{n},\vec{m} \rangle} \left[J_{ab} \vec{S}_{\vec{n},l} \cdot \vec{S}_{\vec{m},l} + \Gamma_{ab} S^z_{\vec{n},l} S^z_{\vec{m},l} + D_{ab} (-1)^{n_x+n_y+l} \left(S^x_{\vec{n},l} S^y_{\vec{m},l} - S^y_{\vec{n},l} S^x_{\vec{m},l} \right) \right] \\ & + \sum_{\langle\langle \vec{n},\vec{m} \rangle\rangle} J'_{ab} \vec{S}_{\vec{n},l} \cdot \vec{S}_{\vec{m},l} + \sum_{\langle\langle\langle \vec{n},\vec{m} \rangle\rangle\rangle} J''_{ab} \vec{S}_{\vec{n},l} \cdot \vec{S}_{\vec{m},l}, \end{aligned} \tag{S2}$$

and the inter-plane (c-axis) coupling is described by

$$H_c = \sum_{\vec{n}} \left[J_c \vec{S}_{\vec{n},1} \cdot \vec{S}_{\vec{n},2} + \Gamma_c S^z_{\vec{n},1} S^z_{\vec{n},2} + D_c (-1)^{n_x+n_y} \left(S^x_{\vec{n},1} S^y_{\vec{n},2} - S^y_{\vec{n},1} S^x_{\vec{n},2} \right) \right] + \sum_{\langle\langle \vec{n},\vec{m} \rangle\rangle} J'_c \vec{S}_{\vec{n},1} \cdot \vec{S}_{\vec{m},2}. \tag{S3}$$

Here $\vec{S}_{\vec{n},l}$ is the vector of isospin operators $S^\alpha = \hbar\sigma^\alpha/2$ with σ^α the spin-1/2 Pauli matrices acting on the isospin doublet on the site $\vec{n} = (n_x, n_y)$ of the lth layer. J_{ab} (J_c) is the in-plane (inter-plane) Heisenberg exchange parameters, Γ_{ab} (Γ_c) is the in-plane (inter-plane) pseudo-dipolar anisotropic exchange, and D_{ab} (D_c) is the in-plane (inter-plane) Dzyaloshinskii-Moriya interaction. We have also introduced longer range exchange couplings, with J' describing the next-nearest-neighbor Heisenberg interaction and J'' the next-next-neighbor. Our exchange parameters are defined for the case

$$\sum_{\langle \vec{n},\vec{m} \rangle} O_{\vec{n}} O_{\vec{m}} = \sum_{\vec{n}} \sum_{\vec{o}=\hat{a},\hat{b}} O_{\vec{n}} O_{\vec{n}+\vec{o}} \tag{S4}$$

where $\hat{a}, \hat{b}$ are the nearest-neighbor vectors within the ab plane.

The classical canted antiferromagnet ground state, about which we compute the magnon dispersion for the 1SIO/1STO SL, is described by the *classical spin configuration*

$$S^x_{\vec{n}} = s(-1)^{n_x+n_y} \cos\psi, \qquad S^y_{\vec{n}} = s \sin\psi, \qquad S^z_{\vec{n}} = 0, \tag{S5}$$

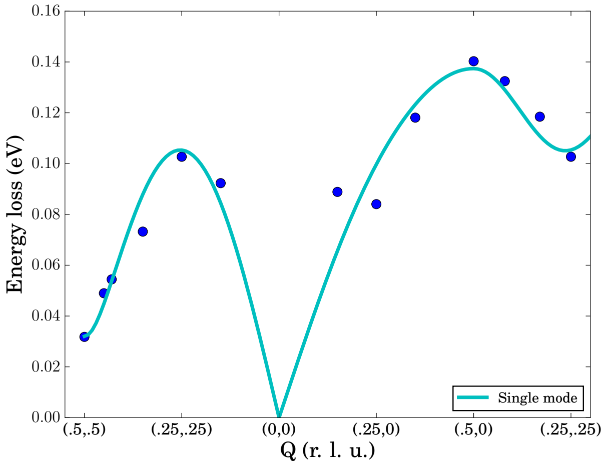

FIG. S12. Fit of the averaged magnon dispersion (cyan line) to that extracted from the RIXS intensity (solid blue points) for 1SIO/STO.

where $s = 1/2$ is the magnitude of the spin and ψ is the canting angle that satisfies $\tan(2\psi) = D_{ab}/J_{ab}$. Application of linear spin wave theory gives a single magnon branch, with dispersion relation dispersion relations

$$E(\vec{k}) = 2\hbar^2 s\sqrt{\bar{B}_{\vec{k}}^2 - \bar{C}_{\vec{k}}^2}. \tag{S6}$$

with

$$\bar{B}_{\vec{k}} = 4\sqrt{J^2 + D^2} - 2J''\left(1 - \gamma_{2\vec{k}}\right) + 2\left(J + \Gamma - \sqrt{J^2 + D^2}\right)\gamma_{\vec{k}} - 2J'\left[1 - \cos(k_x)\cos(k_y)\right], \tag{S7}$$

$$\bar{C}_{\vec{k}} = 2\left(J + \Gamma + \sqrt{J^2 + D^2}\right)\gamma_{\vec{k}}. \tag{S8}$$

where $\gamma_{\vec{k}} = \frac{1}{2}(\cos k_x + \cos k_y)$. A fit of our results to the experimental data for 1SIO/STO, with momentum-dependent scattering intensity accounted for, is shown in Fig. S12.

The classical z Néel antiferromagnet ground state, about which we compute the magnon dispersion for the 2SIO/STO SL, is described by the *classical spin configuration*

$$S^x_{\vec{n},l} = 0, \qquad S^y_{\vec{n}} = 0, \qquad S^z_{\vec{n}} = s(-1)^{n_x+n_y+l}, \tag{S9}$$

where $s = 1/2$ is the magnitude of the spin.

The application of linear spin wave theory yields two magnon branches, with dispersion relations

$$E_{\pm}(\vec{k}) = \hbar^2 s\sqrt{B^2_{\pm,\vec{k}} - X^2_{\pm,\vec{k}} - Y^2_{\pm,\vec{k}}}\ , \tag{S10}$$

with

$$B_{\pm,\vec{k}} = \frac{1}{2}(8J_{ab} + 8\Gamma_{ab} + J_c + \Gamma_c) - 4J''_{ab}(1 - \gamma_{2\vec{k}}) - 4J'_{ab}\left[1 - \cos(k_x)\cos(k_y)\right] - 2J'_c(1 \mp \gamma_{\vec{k}}), \tag{S11}$$

$$X_{\pm,\vec{k}} = \frac{1}{2}(8J_{ab}\gamma_{\vec{k}} \pm J_c), \qquad Y_{\pm,\vec{k}} = -\frac{1}{2}(8D_{ab}\gamma_{\vec{k}} \pm D_c), \tag{S12}$$

The intensity of the two magnon branches as a function of in-plane Q can also be computed from the imaginary part of the low-energy dynamical susceptibility. We use the known approximation [13] to the full form,

$$F_{\pm}(\vec{Q}) = \hbar^2 sV\frac{B_{\pm,\vec{Q}} - X_{\pm,\vec{Q}}}{\sqrt{B^2_{\pm,\vec{Q}} - X^2_{\pm,\vec{Q}} - Y^2_{\pm,\vec{Q}}}}. \tag{S13}$$

A fit of our results to the experimental data, with momentum-dependent scattering intensity accounted for, is shown in Fig. S13. It is clear at each Q one mode is clearly dominate except at (0, 0) where the intensity is at minimum, explaining the lack of two distinct features in our spectra. Further, where there is a mixture of the two modes and significant intensity the separation is below the experimental resolution. Thus, in our spectra we see one feature which has optical or acoustic character depending non-trivially on Q.

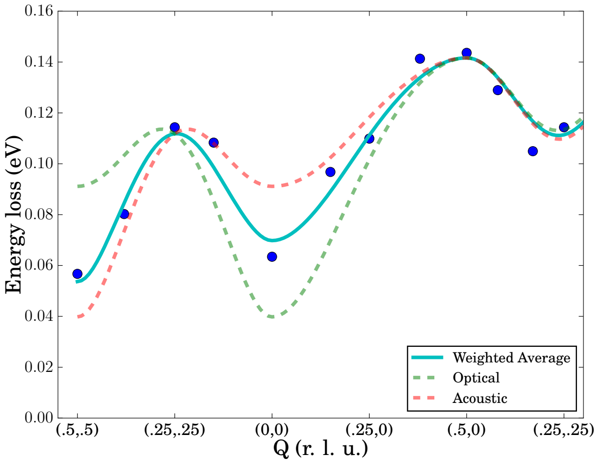

FIG. S13. Fit of the averaged magnon dispersion (cyan line) to that extracted from the RIXS intensity (solid blue points). The optical and acoustic magnon dispersions are calculated within spin wave theory, Eq. (S10), and are averaged to account for their momentum-dependent scattering intensities. Averaging is performed to take into account, to leading order, the energy resolution (35 meV) and weak signal of the experiment.

B. Dispersion Fitting

The exchange coupling parameters were parameterized as before [13, 16] (in units of $\frac{4t^2}{U}$):

$$\begin{aligned} J_{ab} &= \nu_1(t_s^2 - t_a^2) + \nu_2 \cos^2\theta \cos 2\theta \\ J_c &= \nu_1 \cos^4\theta \cos 4\theta \\ \Gamma_{ab} &= 2\nu_1 t_a^2 - \nu_2 \cos^2\theta \cos 2\theta \\ \Gamma_c &= 2\nu_1 \cos^4 \sin^2 2\alpha + 2\nu_2 \cos^2\theta \sin^2\theta \\ D_{ab} &= 2\nu_1 t_s t_a \\ D_c &= \nu_1 \cos^4 \sin 4\alpha \end{aligned} \tag{S14}$$

where $t_s = \sin^2\theta + \frac{1}{2}\cos^2\theta\cos 2\alpha$, $t_a = \frac{1}{2}\cos^2\theta \sin 2\alpha$, $\nu_1 = \frac{3r_1+r_2+2r_3}{6}$, $\nu_2 = \frac{r_1-r_2}{4}$, $r_1 = \frac{1}{1-3\eta}$, $r_2 = \frac{1}{1-\eta}$, and $r_3 = \frac{1}{1+2\eta}$. The remaining couplings, J'_{ab}, J''_{ab}, and J'_c were allowed to freely float after fitting θ. As noted by J. H. Kim *et al.*, these couplings only weakly effect the shape of the dispersion away from the gaps, with no appreciable effect on the observed gaps.

Using these parameters with Eq. (S10), θ and the magnetic bandwidth, W, applied to all couplings were fit with η and α fixed to 0.24 and 8°, respectively. The fit is displayed in Fig. S13 and Fig. S12 with $\theta = 0.231 \pm 0.008\pi$ and $W = 17.0 \pm 0.49$ and $\theta = 0.234 \pm 0.01\pi$ and $W = 20.4 \pm 0.75$ for 2SIO/STO and 1SIO/STO respectively. We note changing to the theoretically predicted $\alpha = 14°$ changes the result for 2SIO/STO to $\theta = 0.222 \pm 0.007\pi$, which would further strengthen the argument for reduced magnetic phase stability.

For the bi-layer, there are two active magnon modes, optical and acoustic, with the $(0.5, 0.5)$ gap dominated by the optical mode, and the (0,0) gap being a mixture of both modes. Where the two modes both have strong intensities their energy separation is small compared to the $\sim$ 100 meV width of the feature and thus we observed only one feature in each spectra. However, the separation at (0, 0) is significant, but the extremely low intensity likely makes the two modes too small to distinguish.

For completeness, we also ran the fit with η fixed with values as low as 0.18 and as high as 0.30, which caused changes to the fit θ value of $\pm$ 0.003π, still within the statistical error of the original fit, showing our assumption of similar η to $Sr_3Ir_2O_7$ is not consequential to the obtained conclusions. Values of α in the range $0 - 20°$ are compatible with the RIXS data while θ always remains below 0.243π ($\theta = 0.26\pi$ for $Sr_3Ir_2O_7$) emphasizing the robustness of our finding of a lowered magnetic phase stability. We thus fix $\alpha = 8°$ based on experimental diffraction data [1].

C. Dimer approach

For $Sr_3Ir_2O_7$ the dispersion and magnon gap can also be described in terms of a bond-operator approach utilizing spin dimers along the c-axis [5], not considered here for several reasons. In this case, the SLs lies firmly within the antiferromagnetic order regime as clearly evidenced by the smaller magnon gaps, whereas the dimer model requires gaps $\geq$ 90 meV. Finally, the sizable total moments observed, on the order of Sr_2IrO_4, also conflict with a dimer picture in this case.

V. ADDITIONAL DATA

A. 1SIO/1STO

Fig. S14 compares data at Q=(0.5, 0) for two 1SIO/1STO samples, original is the data within the manuscript. The manuscript data gives a magnetic excitation energy of 143.1 $\pm$ 7.9 meV, whereas the new data gives 146.6 $\pm$ 3.5 meV, showing the consistency of our results despite the differing elastic line profiles which derives from surface, geometry, and substrate factors.

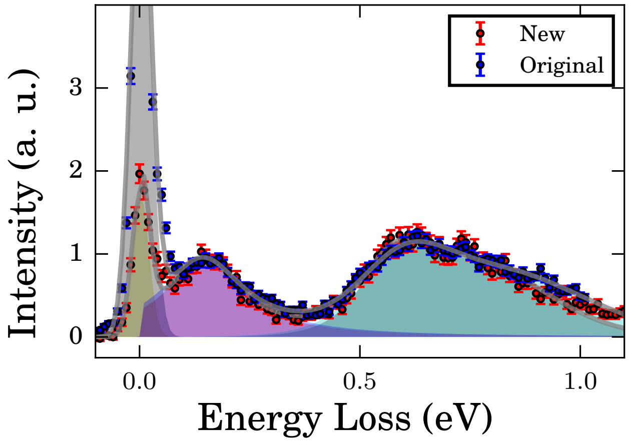

FIG. S14. Fitting curves for Q=(0.5, 0) for two different samples showing consistent magnetic excitations.

B. 2SIO/1STO

RIXS spectra were also taken for a separate 2SIO/1STO sample in order to confirm the change in the magnon gap is due to the heterostructure morphology and not an anomaly. For this purpose we also took several spectra in the vicinity of the magnon gap including (0.50, 0.50), (0.48, 0.48) and (0.45, 0.45). The results are summarized in Figs. S15 and S16 for the original and alternative fitting routines respectively. Fitting of these dispersions gave $\theta = 0.229 \pm 0.005\pi$ and $W = 15.72 \pm 0.38$ and $\theta = 0.228 \pm 0.003\pi$ and $W = 14.12 \pm 0.22$, respectively. Both of these results are well within the error bars for the θ and W found for the 2SIO/1STO sample shown in the manuscript.

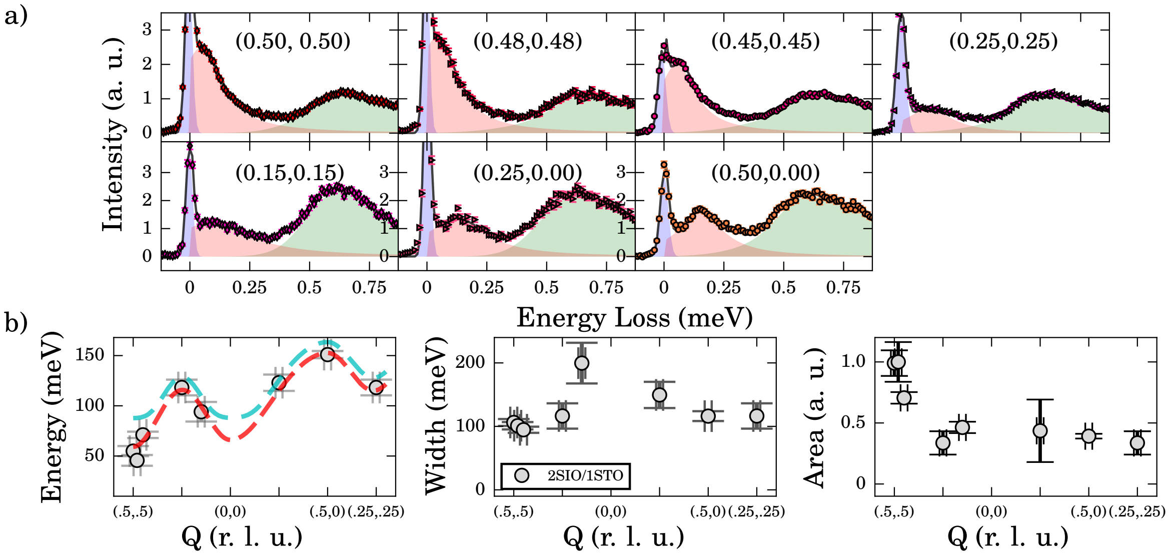

FIG. S15. a) RIXS spectra for new 2SIO/1STO sample with Bose-corrected Lorentzian lineshape. The magnon feature is displayed in red, the excitonic features in green, and the elastic line in blue, while the total of these fitting contributions is a dark grey line. b) Extracted energy loss (left), width (middle), and integrated intensity (right) of the magnon feature across reciprocal space. The dashed red (cyan) line is the magnon dispersion fit ($Sr_3Ir_2O_7$ [13]). The error bars are propagated from the uncertainty in the least-squares fitting routines.

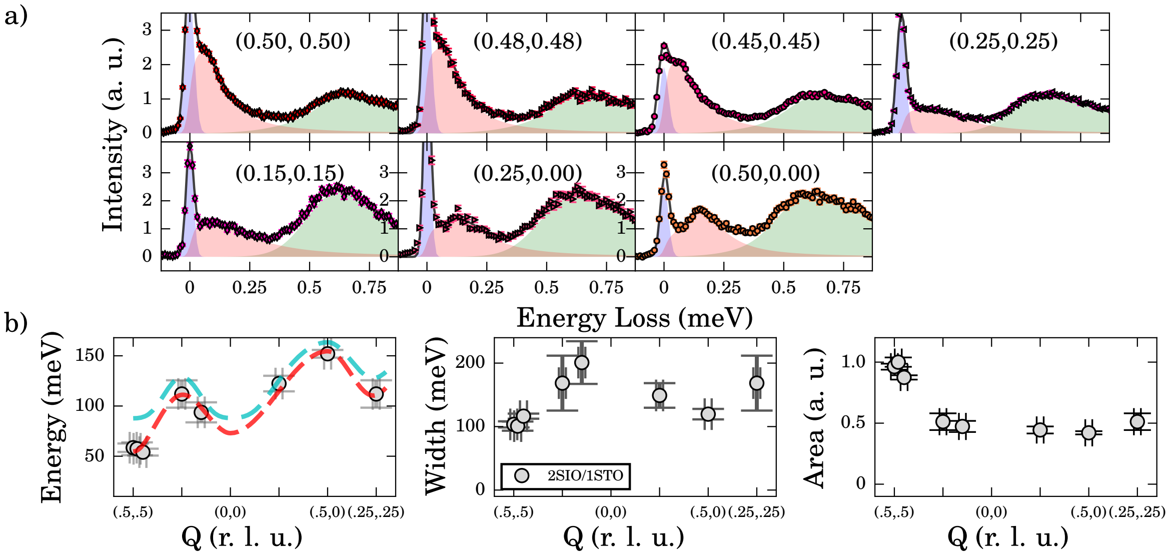

FIG. S16. a) RIXS spectra for new 2SIO/1STO sample with alternative lineshape fitting. The magnon feature is displayed in red, the excitonic features in green, and the elastic line in blue, while the total of these fitting contributions is a dark grey line. b) Extracted energy loss (left), width (middle), and integrated intensity (right) of the magnon feature across reciprocal space. The dashed red (cyan) line is the magnon dispersion fit ($Sr_3Ir_2O_7$ [13]). The error bars are propagated from the uncertainty in the least-squares fitting routines.

[1] J. Matsuno, K. Ihara, S. Yamamura, H. Wadati, K. Ishii, V. V. Shankar, H.-Y. Kee, and H. Takagi, Phys. Rev. Lett. **114**, 247209 (2015).
[2] L. Hao, D. Meyers, H. Suwa, J. Yang, C. Frederick, T. R. Dasa, G. Fabbris, L. Horak, D. Kriegner, Y. Choi, *et al.*, arXiv preprint arXiv:1804.08780 (2018).
[3] J. P. Clancy, N. Chen, C. Y. Kim, W. F. Chen, K. W. Plumb, B. C. Jeon, T. W. Noh, and Y.-J. Kim, Phys. Rev. B **86**, 195131 (2012).
[4] D. Haskel, G. Fabbris, M. Zhernenkov, P. P. Kong, C. Q. Jin, G. Cao, and M. van Veenendaal, Phys. Rev. Lett. **109**, 027204 (2012).
[5] M. Moretti Sala, V. Schnells, S. Boseggia, L. Simonelli, A. Al-Zein, J. G. Vale, L. Paolasini, E. C. Hunter, R. S. Perry, D. Prabhakaran, A. T. Boothroyd, M. Krisch, G. Monaco, H. M. Rønnow, D. F. McMorrow, and F. Mila, Phys. Rev. B **92**, 024405 (2015).
[6] B. M. Wojek, M. H. Berntsen, S. Boseggia, A. T. Boothroyd, D. Prabhakaran, D. F. McMorrow, H. M. Rønnow, J. Chang, and O. Tjernberg, J. Phys. Cond. Matter **24**, 415602 (2012).
[7] M. Björck and G. Andersson, J. Appl. Crystallogr. **40**, 1174 (2007).
[8] J. P. Hill and D. F. McMorrow, Acta Cryst. **A52**, 236 (1996).
[9] S. Boseggia, R. Springell, H. C. Walker, A. T. Boothroyd, D. Prabhakaran, S. P. Collins, and D. F. McMorrow, J. Phys. Cond. Matter **24**, 312202 (2012).
[10] B. J. Kim, H. Ohsumi, T. Komesu, S. Sakai, T. Morita, H. Takagi, and T. Arima, Science **323**, 1329 (2009).
[11] A. M. Glazer, Acta Cryst. A **31**, 756 (1975).
[12] J. Kim, D. Casa, M. H. Upton, T. Gog, Y.-J. Kim, J. F. Mitchell, M. van Veenendaal, M. Daghofer, J. van den Brink, G. Khaliullin, and B. J. Kim, Phys. Rev. Lett. **108**, 177003 (2012).
[13] J. Kim, A. H. Said, D. Casa, M. H. Upton, T. Gog, M. Daghofer, G. Jackeli, J. van den Brink, G. Khaliullin, and B. J. Kim, Phys. Rev. Lett. **109**, 157402 (2012).
[14] W.-G. Yin, X. Liu, A. M. Tsvelik, M. P. M. Dean, M. H. Upton, J. Kim, D. Casa, A. Said, T. Gog, T. F. Qi, G. Cao, and J. P. Hill, Phys. Rev. Lett. **111**, 057202 (2013).
[15] X. Liu, M. P. M. Dean, Z. Y. Meng, M. H. Upton, T. Qi, T. Gog, Y. Cao, J. Q. Lin, D. Meyers, H. Ding, *et al.*, Phys. Rev. B **93**, 241102 (2016).
[16] J. W. Kim, Y. Choi, J. Kim, J. F. Mitchell, G. Jackeli, M. Daghofer, J. van den Brink, G. Khaliullin, and B. J. Kim, Phys. Rev. Lett. **109**, 037204 (2012).
